# Supplementary material for: HIVprotI: an integrated web based platform for prediction and design of HIV proteins inhibitors
Source: J Cheminform. 2018 Mar 9;10:12. doi: 10.1186/s13321-018-0266-y (PMC5845081; doi:10.1186/s13321-018-0266-y)
Supplement: Supplementary file 2 — Additional file 2. Source code of HIVProtI web server. [file 13321_2018_266_MOESM2_ESM.zip › HIVprotI_Source-code/hivprotI/jsme/index.html]

JSME Molecule Editor


# JSME Molecule Editor Distribution

This distribution contains 3 directories:  

- jsme - the actual JSME code - every file in the directory is a JavaScript code optimized for specific browser
- api\_javadoc - the JSME API - please note that although the JSME is in JavaScript, the documentation was generated automatically and therefore follows the Java conventions, anyway it should provide sufficient information to call low-level JSME functions
- jme\_examples - examples of usage of JSME using the JME to JSME migration

- example1
- example2
- example3
- example4

Files contained in the current directory should help you to implement JSME easily:

doc.html provides basic information about JSME implementation into web page - start with this document  

Example pages:  

- JSME\_minimal.html minimalistic example how to incorporate JSME into a web page
- JSME\_test.html Example containing description of various program options
- JSME\_dnd\_demo.html Example illustrating JSME drag-and-drop capabilities
- JSME\_atom\_highlight\_demo.html Example of atom highlighting
- JSME\_smiles\_atom\_highlight.html Example of atom highlighting inside a SMILES
- JSME\_chemical\_resolver\_demo.html JSME can communicate with the Chemical Identifier Resolver service at NIH and automatically retrieve pasted molecules
- JSME\_callback\_and\_star.htmlJavaScript callback for structure modification. Atom marking
- JSME\_resize.html Resize the applet
- JSME\_autoresize.html Resize the applet when the window size changes
- JSME\_depict\_smiles.html JSME in depict mode initialized with a SMILES
- JSME\_depict\_edit\_toggle.html Toggle between depict and edit mode
- JSME\_depict\_action\_callback.html Depict mode with callbacks
- JSME\_parent\_and\_metabolites.html Setup for metabolites generation that uses custom templates
- JSME\_template.html Creation of custom templates

And some other files:  

- license.txt contains text of the BSD license under which the JSME is distributed
- release\_notes.txt provides JSME changelog

If you are using the JSME cite please the following article:  
B. Bienfait and P. Ertl, **JSME: a free molecule editor in JavaScript**, J. Cheminformatics 5:24 (2013)

Happy molecule editing!
